# Dietary Cadmium Exposure in Black Goats: Impacts on Growth Performance, Serum Biochemical and Immune Parameters, Meat Quality, and Tissue Accumulation

**DOI:** 10.3390/ani16193115

**Published:** 2026-10-04

**Authors:** Yang Luo, Bin Yang, Qinyang Jiang

**Affiliations:** 1 College of Animal Science and Technology, Guangxi University, Nanning 530004, China; xinhelu509@hunaas.cn; 2 Hunan Institute of Animal and Veterinary Science, Changsha 410131, China; 3 School of Biological and Chemical Engineering, Zhejiang University of Science and Technology, Hangzhou 310023, China; binyang@zust.edu.cn

**Keywords:** cadmium, growth performance, serum index, rice straw, black goat

## Abstract

Cadmium (Cd) present in soil can accumulate in plants and subsequently affect the safety and quality of animal-derived food products. This study investigated the effects of dietary Cd exposure derived from rice straw on the growth performance, serum biochemical and immune parameters, meat quality, and tissue accumulation of black goats. A 90-day feeding trial was carried out with 42 healthy male black goats at 4 months. Prior to the feeding trial, six goats were slaughtered to establish baseline group, and the remaining 36 goats were randomly assigned to two groups fed with a diet containing Cd-contaminated rice straw (CAD, *n* = 18) or not (CON, *n* = 18). At 30, 60, and 90 d, six goats per group (*n* = 6) were randomly selected and slaughtered for sample collection. The results showed that dietary Cd exposure had no significant effects on growth performance and the indices of heart, liver, spleen, lung, and kidney (*p* > 0.05). Meanwhile, it induced tissue-specific and time-dependent Cd accumulation, with lymph nodes identified as the primary Cd target tissue in goats, followed by kidney and liver (*p* < 0.05). The CAD group exhibited higher concentrations of serum urea nitrogen and interleukin-10, and lower concentrations of serum glucose and creatinine. Fecal cadmium deposition efficiency (CdE) ranged from 95.68% to 97.01% in the CON group and from 68.48% to 88.74% in the CAD group, whereas the CdE in muscle and internal organs remained below 2% in both groups. These findings indicate that fecal excretion constitutes the predominant elimination pathway for ingested Cd, with only a negligible proportion retained in edible tissues and internal organs. However, based on the present findings, we cannot recommend the use of Cd-contaminated rice straw as a feed source for goats, nor can we confirm the safety of meat from exposed animals for human consumption. Further long-term studies, including human health risk assessments, are needed before any recommendations can be made.

## Simple Summary

Cadmium is a heavy metal that can enter the food chain through contaminated soil and crops, potentially affecting animal health and the safety of meat products. This study investigated whether feeding goats with rice straw containing low levels of cadmium influenced their growth performance, serum indicators, and the accumulation of cadmium in different organs. Over a 90-day feeding trial, goats were fed either a normal diet or a diet containing cadmium-contaminated rice straw. The results showed that cadmium exposure did not affect growth performance or organ development, but it did lead to cadmium accumulation in certain tissues, particularly in the kidneys, liver, and lymph nodes. Serum parameters and immune responses were also altered, while meat tenderness and water-holding capacity were improved. Cadmium levels in edible tissues remained within safety limits throughout the trial. However, based on these findings, we cannot recommend the use of cadmium-contaminated rice straw as a feed source for goats, nor can we confirm that meat from exposed animals is safe for human consumption. Further long-term studies, including human health risk assessments, are needed before any recommendations can be made.

## 1. Introduction

Cadmium (Cd) is a non-essential element with no known physiological or biochemical function in animals or humans, and no dietary requirement exists. On the contrary, Cd is one of the most toxic and widespread heavy metal pollutants in the worldwide agricultural ecosystems. Due to industrial emissions, sewage irrigation, and excessive application of phosphate fertilizers, Cd contamination in arable soils has become a global environmental concern, particularly in China [1,2,3]. Unlike organic pollutants, Cd is non-biodegradable and persists in the environment indefinitely, posing long-term risks to ecological safety and human health.

In China, approximately 7% of arable soils exceed the Cd limit. In southern provinces such as Hunan, industrial emissions, mining activities, sewage irrigation, and long-term use of Cd-containing phosphate fertilizers have led to elevated Cd levels in paddy soils. Consequently, Cd concentrations in rice straw can reach 1–3 mg/kg, exceeding the Chinese feed hygiene standard (GB 2762–2022) [4]. Rice (*Oryza sativa* L.) is a staple food crop for more than half of the world’s population, and it has a strong ability to absorb and accumulate Cd from contaminated soils [5,6,7]. Rice straw is a major roughage source for ruminant livestock such as goats, cattle, and sheep. When rice is grown in Cd-contaminated soils, significant amounts of Cd can be translocated to the straw, which then enters the animal food chain when used as feed. This transfer pathway represents a critical link between environmental pollution and food safety.

Cd accumulated in animal tissues can eventually be consumed by humans through meat and milk, leading to chronic Cd poisoning and associated health problems such as renal dysfunction, stroke, cardiovascular diseases and increased cancer risk [8,9,10,11]. Goats are widely raised in southern China due to their strong adaptability and high-quality meat. However, data on Cd transfer to goat products and the associated human health risks are scarce. Therefore, evaluating Cd accumulation in goats fed Cd-contaminated rice straw is essential for food safety. Our previous study on beef cattle demonstrated that dietary Cd exposure through Cd-accumulated maize did not present negative effects to growth performance [12]. Meanwhile Cd is primarily absorbed in the gastrointestinal tract and accumulates mainly in the liver and kidneys, with lower concentrations found in muscle, which caused risks to cattle health and production safety concern [12,13]. Goats are widely raised in southern China due to their strong adaptability and high-quality meat. Whether their production performance of goats would be affected by the Cd contaminated feedstuffs is unclear.

Therefore, the present study was designed to conduct a 90-day feeding trial of dietary Cd exposure by using Cd-contaminated rice straw on growing black goats. The growth performance, organ development, serum biochemical and immune parameters, tissue Cd accumulation, and feces Cd excretion in black goats were measured to investigate the effects of dietary Cd exposure on growing black goats. The results of this study will provide valuable data for assessing the health risks of Cd exposure in goats and establishing safety guidelines for the use of Cd-contaminated agricultural byproducts in animal feed.

## 2. Materials and Methods

### 2.1. Animal Care

The experiment was conducted at Hunan Institute of Animal and Veterinary Science (HIAVS). The protocol and experimental procedures were approved by the Institutional Animal Care and Use Committee (IACUC) of HIAVS (Approval No. HIAVS-IACUC-2025-03).

### 2.2. Animals and Experimental Design

A total of 42 healthy male hybrid black goats (male Nubian black goat crossbreed with female Xiangdong black goat) at 4 months of age (body weight (BW) = 14.10 ± 1.95 kg) were purchased from Liu’an agricultural science and technology comprehensive development Co., Ltd (Liuyang, China). Upon arrival at the experimental base of HIAVS, goats were individually housed in indoor pens (0.7 m by 1.2 m) equipped with fiberglass-reinforced plastic leaky floors. A 5 d adaptation period was implemented to mitigate transport stress, followed by a 90 d feeding trial. Before the feeding trial (0 d), 6 goats were weighed and slaughtered to serve as the baseline (BAS) group. The remaining 36 goats were randomly allocated to one of two dietary treatment groups (*n* = 18 per group) as follows: a control group (CON) fed a normal diet, and a Cd-exposed group (CAD) fed a diet containing Cd-contaminated rice straw. At 30, 60, and 90 days of the experimental period, 6 goats per group were randomly selected, weighed, and slaughtered for sample collection. Therefore, there were six replicates in each group, which provided 80% power (α = 0.05, two-sided) to detect the effects. Throughout the experimental period, goats were individually fed and had free access to fresh water via nipple drinkers. The orts were removed once daily before the morning feeding, and new feed was delivered twice daily at 07:00 and 17:00. The daily amount of feed offered was adjusted based on the previous day’s feed refusals to maintain a residual feed level of approximately 5–10%. Feed and fresh water were available ad libitum. Pens were cleaned daily, and feeders and waterers were disinfected weekly. Goats were fasted for 12 h (with free access to water) before weighing and slaughter.

The experimental rice straw was collected from a normal farmland in Liuyang City for CON group and collected from a farmland with excessive Cd concentration in Zhuzhou City for CAD group. All straws were air-dried and ground to pass through a 1–2 mm sieve using a crusher. The soybean meal, corn, wheat bran, and premixes used in this experiment were provided by Liu’an Agricultural Science and Technology Development Co., Ltd. All ingredients were thoroughly mixed according to the experimental feed formula and pressed into pellets (1–1.5 cm in diameter) using a feed pellet machine. The pelleted feed was then stored in sealed feed bags until use. The ingredients and nutrient levels of diet are shown in Table 1.

### 2.3. Growth Performance Analysis

During the feeding trial, the amount of feed offered and refused was recorded daily for each goat. Average daily feed intake (ADFI, g/d) was calculated by dividing total feed intake by the number of experimental days:

$$\mathrm{ADFI}(g/d)=\frac{\mathrm{Total feed intake}(g)}{\mathrm{Experimental duration}(d)}$$

The BW and body size (including body height, body side length, chest circumference, abdominal circumference, cannon circumference and testicular circumference) of goat were measured after a 12 h fasting period. Initial body weight (IBW) and final body weight (FBW) were determined as the means of two consecutive fasting weights recorded before and at the end of the trial, respectively. Average daily gain (ADG, g/d) was calculated using the following equation:

$$\mathrm{ADG}(g/d)=\frac{\mathrm{FBW}(g)-\mathrm{IBW}(g)}{\mathrm{Experimental duration}(d)}$$

### 2.4. Feed Nutrition Analysis

Feed samples were collected monthly, dried in an oven at 65 °C for 48 h, and ground to allow passage through a 1 mm sieve before nutrition analysis. The dry matter (DM; method 924.05; AOAC,1990), crude protein (CP; method 988.05; AOAC, 1990), calcium (Ca; method 935.13; AOAC, 1990) and phosphorus (P; method 964.06; AOAC 1990) of feed samples were determined according to the method described [14]. The neutral detergent fiber (NDF) and acid detergent fiber (ADF) contents were determined using Ankom filter bags and fiber analyzer equipment (Ankom Technology, New York, USA) following the method of Van Soest et al. [15].

### 2.5. Serum Indices Analysis

Before slaughter, 10 mL of blood was collected via jugular vein puncture from fasted goats using standard vacuum clotting collection tubes. Blood samples were centrifuged at 3000× *g* at room temperature for 15 min to obtain the serum and then stored at −80 °C for biochemical and immune indices analysis. Serum biochemical indices including total protein (TP), total cholesterol (TC), triglycerides (TGs), uric acid (UA), fasting blood glucose (GLU), high-density lipoprotein cholesterol (HDL-C), low-density lipoprotein cholesterol (LDL-C), gamma-glutamyl transpeptidase (GGT), alkaline phosphatase (ALP), creatinine (CR), blood urea nitrogen (BUN), aspartate transaminase (AST), alanine aminotransferase (ALT), albumin (ALB), and globulin (GLB) were measured using an automatic biochemical analyzer (BS-280; Mindray Medical International Co., Ltd., Shenzhen, China) with the commercial kits (Nanjing Jiancheng Bioengineering Institute, Nanjing, China). Immunological indices, including immunoglobulin A (IgA), immunoglobulin G (IgG), immunoglobulin M (IgM), complement C3, complement C4, interleu-kin-2 (IL-2), interleukin-6 (IL-6), and tumor necrosis factor-α (TNF-α), were determined using commercial kits (Shanghai Enzyme-linked Biotechnology Co., Ltd., Shanghai, China) with a microplate reader (RT-6100, Rayto, Shenzhen, China).

### 2.6. Slaughter Performance and Organ Indices Analysis

After a 24 h fast and 2 h water deprivation, live body weight was measured prior to slaughter. Exsanguination was performed via cervical severance. Subsequently, the head, hooves, and tail were removed, followed by skinning and evisceration (removal of the abdominal and thoracic contents). The visceral organs (heart, liver, spleen, lungs, and kidneys) were excised and weighed. Finally, the carcass weight was recorded. Data were applied to calculate dressing percentage = (carcass weight/live weight) × 100%; organ indices = (organ weight/live weight) × 100%.

### 2.7. Muscle Quality Analysis

Immediately after slaughter, muscle samples were obtained from the longissimus dorsi. The pH values of muscle samples were recorded at 45 min post-slaughter. Muscle color was evaluated using a colorimeter (SCQ-1A, Beijing, China) following the method of Qin et al. [16]. Muscle samples were trimmed to a thickness of 3 cm and placed on a flat surface. A random point on the exposed muscle area was selected for color measurement; then, the sample was rotated by 45° to be measured again. This procedure was repeated to obtain three readings per point. Three points were measured per sample, and the mean value was calculated.

To determine muscle drip loss, samples were trimmed along the fiber direction into strips measuring 5 cm × 3 cm × 2 cm. Each strip was suspended by an S-shaped hook in-side a disposable transparent plastic cup and stored at 0–4 °C for 24 h. After storage, the surface moisture was gently blotted dry with filter paper, and the sample weight was recorded using an analytical balance. Drip loss was calculated according to the method of Zhang et al. [17].

To determine muscle cooking loss, approximately 30 g of muscle sample was placed in a 15 cm × 10 cm plastic storage bag and heated in a water bath at 100 °C for 45 min. Then, the sample was cooled at room temperature for 20 min, and the final weight was measured. Cooking loss was calculated as the previously described literature [17].

To determine shear force, samples were placed in a constant-temperature water bath at 80 °C for 1 h, followed by cooling at room temperature for 20 min. A circular sampler was used to obtain a core parallel to the muscle fiber orientation, which was then trimmed into blocks measuring 1.5 cm × 1.0 cm × 1.0 cm. Shear force was measured using a muscle tenderness analyzer (CLM-3B, Tenovo International Co., Limited, Beijing, China) following the procedure described by Zhang et al. [17].

### 2.8. Cadmium Analysis

All tissue samples (longissimus dorsi, foreshank, hindshank, intercostal meat, mesenteric lymph nodes and skin) were immediately frozen in liquid nitrogen and stored at −80 °C until analysis of Cd content. About 0.20 g of the sample was precisely weighed and transferred into a polytetrafluoroethylene microwave digestion tube. After adding 10 mL of concentrated nitric acid, the mixture was shaken thoroughly for 1 min and then placed the tube in a 120 °C reaction furnace for 20 min of pre-digestion, until a small amount of yellow smoke appeared from the vessel. Then, 5 mL of high-purity concentrated nitric acid was added and digested in the microwave digestion system. Once digestion was finished, the vessel was moved to a 180 °C acid evaporator and heated for 30 m to reduce the solution volume to approximately 1 mL. The resulting clear solution was diluted to 50 mL with ultrapure water, shaken for 1 min, and filtered through a 0.22 µm filter membrane. Finally, the Cd concentration in the filtrate was measured using ICP-MS (Agilent 7900, USA) at Shanghai Youxuan Biotechnology Co., Ltd (Shanghai, China).

### 2.9. Cadmium Deposition Rate Analysis

On days 30, 60, and 90 of the experiment, total feces were collected and weighed from each goat; approximately 50 g of fecal subsample was taken using a T-type sampler for Cd concentration measurement. The remaining feces were weighed to calculate the Cd deposition rate. The cadmium deposition efficiency (CdE) for each tissue or feces was calculated using the following formula:

*CdE* (%) = [(*C*1 × *M*1) − *Z*0]/(*C*2 × *M*2) × 100

where

*C*1: cadmium concentration in the tissue or feces (μg/kg);

*M*1: total weight of the tissue or feces (kg);

*Z*0: baseline cadmium content in the corresponding tissue or feces from the baseline group (μg);

*C*2: cadmium concentration in the feed (μg/kg);

*M*2: cumulative total feed intake over the corresponding period (kg).

After all the calculations of CdE for organs and excretions were completed, we used the R software (version 4.5.1, R Core Team, 2025) to plot all the data.

### 2.10. Statistical Analysis

All experimental data were analyzed using IBM SPSS Statistics 20 software (IBM Corp, Armonk, NY, USA). Normality tests were performed on growth performance, organ indices, serum parameters, meat quality, and tissue Cd concentrations, and some variables deviated from normality. A generalized linear model (GLM) was therefore used to evaluate the effects of diet (CON vs. CAD), time (30, 60, and 90 days), and their interaction. The BAS group was presented for display only and was not included in the statistical analysis. Post hoc comparisons among group means were performed using Tukey’s HSD test. For CdE, *t*-test was used to calculate the difference between CON vs. CAD at 30, 60, and 90 days. Statistical significance was set at *p* < 0.05, and trends were considered at 0.05 ≤ *p* < 0.10.

## 3. Results

### 3.1. Effects of Dietary Cadmium Exposure on the Growth Performance of Black Goats

As shown in Table 2, all growth performance indices were significantly increased with feeding time (*p* < 0.01). Cd exposure increased body side length and body height of goat (*p* < 0.05). The interaction effect of Cd exposure and feeding time was not significant for any index (*p* > 0.05). No significant difference was observed between the CON and CAD groups at any time point.

### 3.2. Effects of Dietary Cadmium Exposure on the Organ Indices of Black Goats

The effects of Cd exposure on organ indices of black goats are presented in Table 3. Cd exposure tended to increase liver index and kidney index (0.05 < *p* < 0.10). Feeding time affected all organ indices (*p* < 0.01) except heart index, while the interaction effect of diet and time was not significant for any index (*p* > 0.05).

### 3.3. Effects of Dietary Cadmium Exposure on the Serum Biochemical and Immune Indices of Black Goats

The effects of dietary cadmium exposure on serum biochemical and immune indices of black goats are shown in Table 4 and Table 5, respectively. Cd exposure significantly affected the concentrations of serum GLU (*p* = 0.033), CR (*p* < 0.01), and BUN (*p* = 0.013) and tended to affect the concentration of AST/ALF (*p* < 0.01), while no significant effects were observed on other indices (*p* > 0.05). Time showed significant effects on most indices (*p* < 0.05), except for the concentrations of TG, UA, GGT, and ALP. Meanwhile, no significant interaction effects of diet and time were detected for biochemical parameters (*p* > 0.05) except for the concentration of GLU (*p* < 0.01). Compared to CON group, the concentration of GLU decreased at 30 d, and the concentration of CR decreased at both 30 and 60 d in CAD group (*p* < 0.05), while the concentrations of UREA and AST/ALT of CAD group were increased at 60 d when compared to CON group (*p* < 0.05).

For the immune indices, Cd exposure increased the serum concentration of IgG significantly (*p* = 0.021). All immune indices were significantly increased with the increasing feeding time (*p* < 0.001), while no interaction effect of diet and time was observed.

### 3.4. Effects of Dietary Cadmium Exposure on the Slaughter Performance and Meat Quality of Black Goats

The effects of dietary cadmium exposure on slaughter performance and meat quality traits of longissimus dorsi muscle are presented in Table 6. Cd exposure significantly decreased drip loss, cooking yield and shear force (*p* < 0.05) and tended to increase and decrease pH_45min_ (*p* = 0.094). The dressing percentage, loin muscle, color (L*, a*and b* value), drip loss, cooking yield, shear force, and pH_45min_ were affected by increasing feeding time (*p* < 0.05). The water loss rate of muscle tended to be affected by feeding time (0.10 < *p* < 0.05). The shear force was further affected by the interaction of dietary Cd exposure and feeding time. Compared to CON group, the shear force was significantly decreased in CAD group at 90 d (*p* < 0.05).

### 3.5. Effects of Dietary Cadmium Exposure on the Tissue-Specific and Time-Dependent Cadmium Accumulation of Black Goats

The Cd concentrations in 12 different tissues of black goats are presented in Table 7. Among all tissues in goats of CAD group, the highest Cd concentration was observed in mesenteric lymph nodes (102.78 μg/kg) at 90 d, followed by kidney (78.67 μg/kg) and liver (75.27 μg/kg) at 90 d. Among the meat in different parts, the Cd concentrations were increased to 56.67, 46.70, 22.05, and 24.71 μg/kg in goats after feeding CAD diet.

Cd exposure significantly affected the Cd concentration in all measured tissues except skin (*p* < 0.01). Feeding time affected the Cd concentration in all tissues beside lung, spine, skin, hindquarter, intercostal muscles and longissimus dorsi (*p* < 0.01). Significant interaction effects of Cd exposure and feeding time were found in heart, spleen, lung, testis, lymph nodes, and picnic shoulder (*p* < 0.05). The Cd concentrations were increased in varying degrees in all tissues after Cd exposure. Compared to CON group, the Cd concentrations of testis significantly increased since 30 d of the feeding trail (*p* < 0.05). The Cd concentrations of heart in CAD group were temporarily increased at 30 d, and that of lung and longissimus dorsi were temporarily increased at 60 d (*p* < 0.05), compared to CON group at the corresponding time point. Additionally, the Cd concentrations of liver, spleen, kidney, testis, lymph nodes, picnic shoulder, and hindquarter were increased at 90 d, when compared to CON group at 90 d (*p* < 0.05).

### 3.6. Effects of Dietary Cadmium Exposure on the Cadmium Deposition Rate of Black Goats

**Figure 1:**
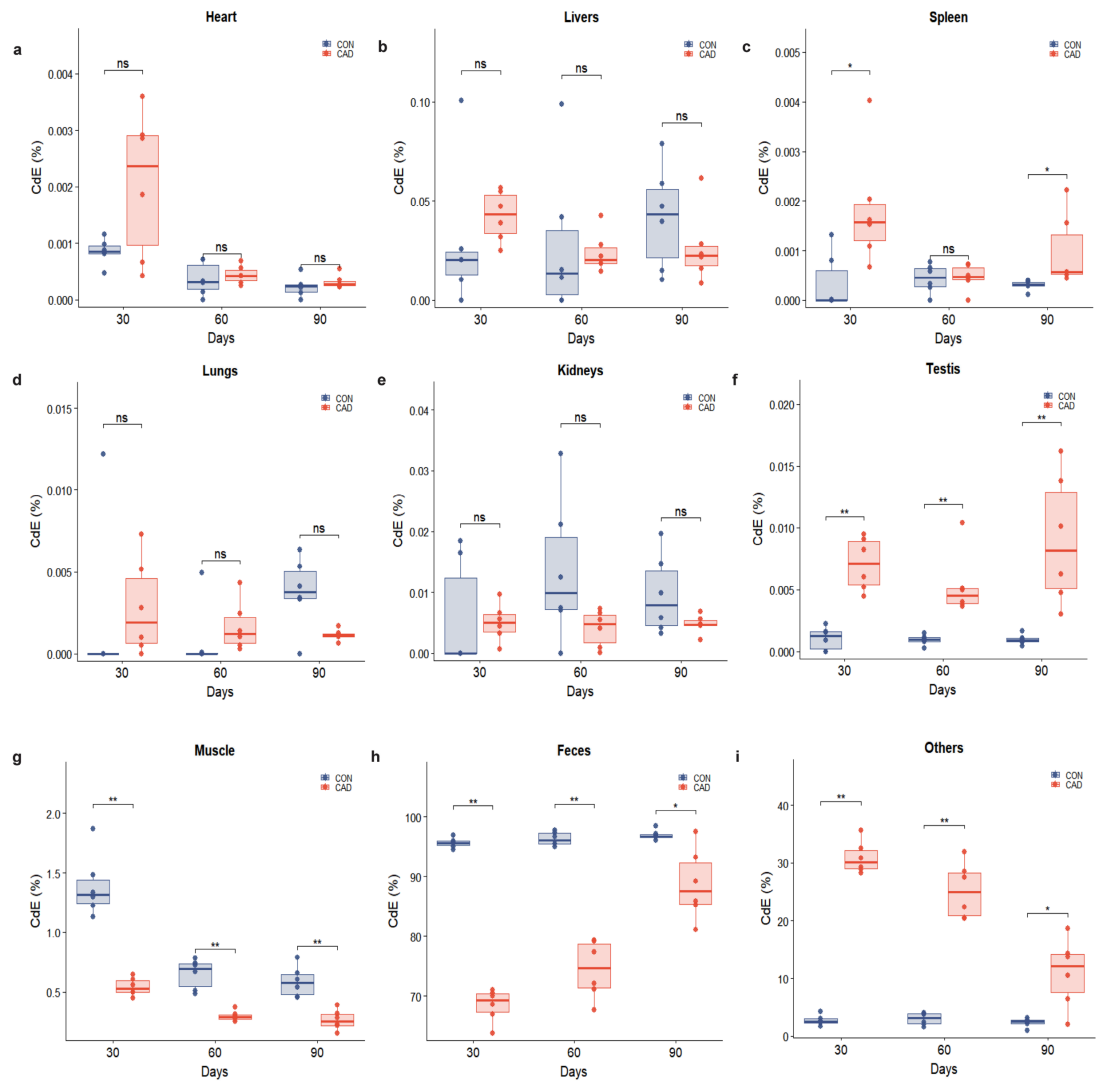
The deposition rate of cadmium in black goats. (**a**–**i**) CdE (%). In the heart, liver, spleen, lungs, kidneys, testicles, muscle, feces, and other parts (such as blood, bones, hair, etc.), data are presented as mean ± SEM (*n* = 6). * Means were significantly different in comparison with the CON group (* *p* < 0.05, ** *p* < 0.05, and ns > 0.05). Blue represents CON, and red represents CAD.

Figure 1 presents the CdE in nine parts of black goats. At each time point, no significant differences were observed between the CON and CAD groups for the heart, liver, lungs, or kidneys (*p* > 0.05). On days 30 and 90, the CdE in the lungs of the CAD group was significantly higher than that of the CON group (*p* < 0.05). Throughout the experimental period, the CdE in the testicular tissue of the CAD group was consistently and significantly higher than CON group (*p* < 0.05). In contrast, the CdE in the muscle tissue of the CAD group was significantly lower than CON group, and both groups exhibited a declining trend over time. Notably, the CdE values in these tissues remained very low, ranging from 0 to 1.87%. In terms of fecal excretion, fecal CdE in the CON group was 95.68%, 96.36%, and 97.01% at days 30, 60, and 90, respectively, while that in the CAD group was 68.48%, 74.48%, and 88.74%, respectively. At each sampling point, the CdE of feces in the CAD group was significantly lower than CON group (*p* < 0.05). For other tissues (such as blood, bone, hair, etc.), the CdE in the CAD group was significantly higher than CON group at all sampling time points (*p* < 0.05).

## 4. Discussion

### 4.1. Growth Performance Analysis in Black Goats

In the present study, dietary Cd exposure from contaminated rice straw had no negative effect on the growth performance, and the Cd concentration in all 13 edible and nonedible tissues of CAD goats did not exceed the maximum residue limits set by the Chinese National Food Safety Standard (GB 2762-2022: 0.5 mg/kg for liver and kidney, 0.1 mg/kg for muscle). These all suggested the tolerance of goat to low-level dietary Cd exposure. The body size in the CAD group was numerically higher than that in the CON group, with the main effect of diet being significant for body-side length. Interestingly, this trend is in stark contrast to previous studies that reported significant growth inhibition in animals exposed to Cd [18,19]. This discrepancy can be primarily attributed to differences in exposure dose and chemical form of Cd. Most existing studies used high doses of inorganic Cd salts such as CdCl_2_ to induce acute toxicity, which do not reflect the actual exposure scenario where animals are chronically exposed to low to moderate levels of Cd bound to plant matrices. In our study, the Cd concentration in the experimental diet was 1.61 mg/kg dry matter, which is above the Chinese national feed safety limit (0.5 mg/kg) but far below the doses used in acute toxicity studies (5–100 mg/kg body weight). Low Cd exposure (0.3 mg/kg CdCl_2_) was reported to cause a slight and nonsignificant numeric increase in mice [20]. Similar results were also observed in beef cattle fed with a diet containing Cd-accumulated maize [12]. Furthermore, Cd in rice straw exists primarily in complexed forms with cellulose, lignin, and phytochelatins, which may have significantly lower bioavailability [18,19] than inorganic Cd salts [21]. On the other hand, exposure time will affect the toxicity of Cd. The present study carried for 90 d might be short to observe the dietary Cd exposure to growth performance.

### 4.2. Serum Biochemical and Immune Indices Analysis in Black Goats

While this does not mean the CAD has no effect on the health of the goats, the increased level of anti-inflammatory IL-10 might represent a compensatory mechanism to counteract Cd-induced oxidative stress and inflammation. Kidney and liver are the target organs for Cd toxicity. Among edible tissues, kidney and liver showed highest Cd accumulation. The decreased liver weight is a well-documented response to Cd exposure damage [22]. While increased liver weight was observed in rats along with hepatomegaly after 6.5 mg/kg CdCl_2_ injection [23], the numerically higher liver index in the CAD group may indicate a potential risk of liver enlargement; however, no universally accepted threshold for goat liver index is currently available. Further histopathological examination is needed to confirm whether hepatomegaly occurred. Among the liver function indices, the increased AST/ALT ratio in the CAD group suggested potential liver damage, consistent with previous studies reporting that an elevated AST/ALT ratio is associated with hepatocellular injury in ruminants [24]. On the other hand, the glucose concentration change pattern in CAD goats was similar with our previous study in beef cattle fed with Cd diet [12]. The decreased glucose concentration at 30–40 d after Cd diet feeding might suggest an inhibition of Cd to liver glucogenesis. While further study on the glucogenesis-related enzyme is also needed for confirmation, the elevated BUN concentration in the CAD group suggested a potential risk of Cd-induced renal impairment, as increased BUN has been commonly used as an indicator of reduced renal function in livestock [25]. Thus, though the Cd accumulation is low, it showed potential health risks to liver and kidney. Interestingly, we found lymph nodes had highest Cd accumulation among the 13 tissues of CAD goats, which was in contrast to a previous study that observed very little accumulation in lymph nodes [26]. This suggested a new target tissue for Cd toxicity and needed further concern in ruminants. To be noted, significant interaction effects between Cd exposure and feeding time were observed in seven tissues, indicating that the Cd accumulation depends not only on exposure dose but also on exposure duration. Thus, a long-term feeding trail is further needed to investigate the long-term effect of dietary Cd exposure.

### 4.3. Cadmium Accumulation and Deposition Rate in Black Goats

In cases of chronic Cd poisoning, approximately 8% of the ingested Cd is absorbed through the gastrointestinal tract [27]. In the present study, we calculated the CdE in various tissues and feces of black goats. Compared with absolute Cd concentration, CdE serves as a more informative indicator for elucidating the tissue-specific deposition pattern of Cd. Our results demonstrated that fecal excretion constitutes the main way for Cd elimination in black goats. However, this process is closely associated with both the dietary Cd concentration and the duration of exposure. Notably, when the dietary Cd concentration was 1.61 mg/kg, an intriguing pattern emerged: over time, CdE in feces increased significantly, whereas CdE in muscle tissues decreased markedly. This may reflect a gradual downregulation of intestinal Cd absorption as an adaptive homeostatic response to sustained exposure. Furthermore, when the dietary Cd concentration remained below 0.5 mg/kg, CdE in feces consistently exceeded 95%, while CdE in the body remained below 5%. Further supports the view that under low-level exposure, the vast majority of ingested Cd passes through the gastrointestinal tract unabsorbed. The tolerance mechanism under long-term low-dose Cd exposure needed further investigation.

### 4.4. Slaughter Performance and Meat Quality Analysis in Black Goats

Interestingly, dietary Cd exposure unexpectedly improved meat yield, juiciness, and tenderness, as reflected by reduced drip loss and shear force, which contrasts with our previous beef cattle study [12] and with the typical Cd-induced oxidative stress and inflammation that impair meat quality [28,29], such as decreased pH_45min_ and increased drip loss in chicken breast [30]. These discrepancies suggest species-, dose-, or duration-dependent effects. A plausible mechanism is that low-dose Cd induces mild oxidative stress, reducing lipid oxidation and preserving membrane integrity to lower drip loss, while disturbing Ca^2+^ homeostasis to activate calpain-mediated proteolysis and reduce shear force; altered intramuscular fat may also contribute [31,32]. These hypotheses require further validation. Additionally, the potential toxicity to human health from cadmium residues in animal meat products remains a concern, and this issue will be further investigated in future studies.

## 5. Conclusions

In conclusion, dietary Cd exposure from contaminated rice straw did not impair growth performance or organ development in growing black goats, but induced tissue-specific Cd accumulation, mainly in lymph nodes, kidneys, and liver. Fecal excretion was the predominant elimination pathway. Although Cd levels in edible tissues remained below national safety limits during the 90-day trial, long-term toxicity was needed to test in further experiment.

## Figures and Tables

**Table 1:** Ingredients and nutrient levels of basal diet.

Items	CON ^3^	CAD ^4^
Ingredients (fresh basis, %)		
Corn	30	30
Soybean meal	12	12
Wheat bran	4	4
Straw	50	50
Premix ^1^	4	4
Total	100	100
Nutrient components ^2^ (DM basis, %)		
DM (fresh basis)	86.59	86.42
Digestible energy (MJ/kg)	10.23	10.23
CP (%)	10.74	10.09
NDF (%)	43.88	41.26
ADF (%)	18.44	15.01
Ca (%)	2.25	1.67
P (%)	0.34	0.33
Cd (mg/kg)	0.38	1.61

^1^ The premix per kilogram diet provided 112,000–160,000 IU vitamin A, 27,000–75,000 IU vitamin D3, >720 IU vitamin E, 280–500 mg copper, 610–1500 mg zinc, 9–10 mg selenium, 11–30 mg cobalt, 17–50 mg iodine, and 320–1500 mg manganese, >18.5% calcium, >0.6% phosphorus, >4.2% sodium, >1.5% magnesium, >0.9% potassium. ^2^ Digestible energy was calculated and other nutrient components were measured. GE = digestible energy; DM = dry matter; CP = crude protein; NDF = neutral detergent fiber; ADF = acid detergent fiber; Ca = calcium; P = phosphorus; Cd = cadmium. ^3^ CON represents a control group fed a normal diet. ^4^ CAD represents a Cd-exposed group fed a diet containing Cd-contaminated rice straw.

**Table 2:** Growth performance of black goats at 30, 60, and 90 days of cadmium exposure.

Items	BAS			CON with Feeding Time, d				CAD with Feeding Time, d			SEM	*p*-Value		
	(0 d)			30	60	90		30	60	90		Diet	Time	Diet × Time
Final body weight (kg)	12.68			13.33 ^c^	21.16 ^b^	27.60 ^a^		14.34 ^c^	20.43 ^b^	28.62 ^a^	0.868	0.544	<0.010	0.521
Average daily gain (g)	0.00			24.89 ^c^	123.69 ^ab^	120.76 ^ab^		53.94 ^bc^	96.94 ^abc^	144.04 ^a^	19.742	0.601	<0.010	0.313
Body height (cm)	49.17			47.83 ^c^	50.58 ^c^	58.33 ^ab^		49.00 ^c^	53.08 ^bc^	61.17 ^a^	1.260	0.044	<0.010	0.784
Body side length (cm)	50.17			49.83 ^d^	53.83 ^bcd^	60.17 ^ab^		51.33 ^cd^	57.67 ^abc^	62.83 ^a^	1.508	0.038	<0.010	0.744
Chest circumference (cm)	52.83			51.83 ^c^	61.67 ^b^	69.33 ^a^		52.93 ^c^	62.42 ^b^	69.50 ^a^	1.086	0.454	<0.010	0.910
Abdominal circumference (cm)	55.92			63.53 ^b^	75.25 ^a^	80.92 ^a^		64.37 ^b^	75.58 ^a^	81.17 ^a^	1.852	0.757	<0.010	0.986
Cannon circumference (cm)	7.05			5.98 ^c^	7.48 ^ab^	8.80 ^a^		6.78 ^bc^	7.28 ^bc^	8.68 ^a^	0.311	0.530	<0.010	0.220
Testicular circumference (cm)	15.95			14.68 ^b^	18.43 ^ab^	21.45 ^a^		15.63 ^b^	17.92 ^ab^	20.87 ^a^	0.994	0.951	<0.010	0.687

^a–d^ Values with different superscript letters in the same row indicate significant differences (*p* < 0.05) and no shoulder notesindicated no significant differences (*p* > 0.05).

**Table 3:** Organ indices of black goats at 30, 60, and 90 days of cadmium exposure.

Items	BAS		CON with Feeding Time, d					CAD with Feeding Time, d			SEM	*p*-Value		
	(0 d)		30	60	90			30	60	90		Diet	Time	Diet × Time
Heart index	0.46		0.41	0.42	0.41			0.4	0.39	0.43	0.018	0.618	0.720	0.389
Liver index	2.09		2.05 ^ab^	1.86 ^bc^	1.55 ^c^			2.31 ^a^	1.96 ^abc^	1.67 ^bc^	0.104	0.068	<0.01	0.710
Spleen index	0.19		0.16 ^ab^	0.13 ^ab^	0.10 ^b^			0.17 ^a^	0.09 ^b^	0.11 ^ab^	0.018	0.889	<0.01	0.321
Lung index	1.40		1.23 ^ab^	1.00 ^b^	0.86 ^b^			1.46 ^a^	0.92 ^b^	0.95 ^b^	0.091	0.281	<0.01	0.230
Kidney index	0.49		0.41 ^ab^	0.34 ^bc^	0.30 ^c^			0.47 ^a^	0.37 ^bc^	0.31 ^c^	0.020	0.085	<0.01	0.503

^a–c^ Values with different superscript letters in the same row indicate significant differences (*p* < 0.05) and no shoulder notesindicated no significant differences (*p* > 0.05).

**Table 4:** Serum biochemical indices of black goats at 30, 60, and 90 days of cadmium exposure.

Items ^1^	BAS		CON with Feeding Time, d				CAD with Feeding Time, d			SEM	*p*-Value		
	(0 d)		30	60	90		30	60	90		Diet	Time	Diet × Time
TP (g/L)	58.77		64.10	64.97	63.69		65.27	65.24	63.62	1.831	0.761	0.775	0.929
TC (mmol/L)	3.07		1.50	1.50	1.35		1.40	1.38	1.30	0.110	0.327	0.539	0.963
TG (mmol/L)	0.48		0.36	0.43	0.38		0.33	0.28	0.3	0.057	0.068	0.967	0.512
UA (umol/L)	5.04		4.93	5.01	4.71		4.6	4.81	4.85	0.427	0.717	0.911	0.871
GLU (mmol/L)	5.03		2.50 ^a^	2.16 ^ab^	2.58 ^a^		1.60 ^b^	2.26 ^a^	2.50 ^a^	0.166	0.033	0.022	<0.01
HDL-C (mmol/L)	1.45		0.92	0.87	0.87		0.88	0.84	0.76	0.056	0.182	0.290	0.804
LDL-C (mmol/L)	1.36		0.50	0.53	0.43		0.45	0.47	0.47	0.057	0.688	0.679	0.713
GGT (U/L)	44.77		50.09	53.96	52.04		51.17	44.65	51.00	3.915	0.337	0.859	0.274
ALP (U/L)	242.38		242.47	365.84	278.28		234.52	363.34	336.31	70.85	0.785	0.089	0.904
CR (μmol/L)	65.70		57.64 ^ab^	55.28 ^ab^	67.18 ^a^		49.22 ^b^	48.70 ^b^	59.55 ^ab^	3.187	<0.01	<0.01	0.940
BUN (mmol/L)	5.89		4.88 ^ab^	3.67 ^b^	5.41 ^ab^		5.43 ^a^	4.89 ^ab^	6.29 ^a^	0.424	0.013	<0.01	0.641
AST (U/L)	102.12		78.72	76.58	87.89		89.86	84.95	89.59	0.433	<0.01	<0.01	0.670
ALT (U/L)	19.96		17.73 ^b^	24.26 ^a^	24.97 ^a^		17.02 ^b^	24.22 ^a^	19.96 ^ab^	1.613	0.149	<0.01	0.349
AST/ALT	5.42		4.69 ^ab^	3.11 ^c^	3.70 ^abc^		5.55 ^a^	3.80 ^a^	5.24 ^a^	0.433	<0.01	<0.01	0.670
ALB (g/L)	22.43		22.45 ^b^	25.05 ^a^	26.42 ^a^		22.04 ^b^	25.01 ^a^	26.94 ^a^	0.646	0.963	<0.01	0.783
GLB (g/L)	36.34		41.66	39.92	37.28		43.23	40.23	36.68	1.781	0.768	0.014	0.817
A/G	0.62		0.55 ^bc^	0.63 ^ab^	0.72 ^a^		0.52 ^c^	0.63 ^ab^	0.74 ^a^	0.030	0.979	<0.01	0.705

^1^ TP = total protein; TC = total cholesterol; TG = triglyceride; UA = uric acid; GLU = glucose; HDL-C = high-density lipoprotein cholesterol; LDL-C = low-density lipoprotein cholesterol; GGT = γ-glutamyl transferase; ALP = alkaline phosphatase; CR = creatinine; UREA = blood urea nitrogen (BUN); AST = aspartate aminotransferase; ALT = alanine aminotransferase; ALB = albumin; GLB = globulin; A/G = ALB/GLB ratio. ^a–c^ Values with different superscript letters in the same row indicate significant differences (*p* < 0.05) and no shoulder notesindicated no significant differences (*p* > 0.05).

**Table 5:** Serum immune indices of black goats at 30, 60, and 90 days of cadmium exposure.

Items ^1^	BAS		CON with Feeding Time, d				CAD with Feeding Time, d			SEM	*p*-Value		
	(0 d)		30	60	90		30	60	90		Diet	Time	Diet × Time
IgA (μg/mL)	142.27		195.64 ^c^	229.70 ^ab^	249.34 ^ab^		204.57 ^bc^	243.12 ^ab^	282.77 ^a^	12.17	0.066	<0.01	0.629
IgG (mg/mL)	4.55		6.08 ^cd^	7.02 ^bc^	9.33 ^a^		5.72 ^d^	6.78 ^bcd^	7.88 ^ab^	0.353	0.021	<0.01	0.270
IgM (μg/mL)	1253.17		1481.97 ^bc^	1912.86 ^a^	1779.24 ^ab^		1351.32 ^c^	1719.19 ^ab^	1886.82 ^a^	91.45	0.337	<0.01	0.325
C3 (μg/mL)	28.29		39.96 ^c^	48.03 ^ab^	51.14 ^ab^		41.31 ^bc^	46.50 ^abc^	56.40 ^a^	2.276	0.364	<0.01	0.402
C4 (μg/mL)	6.95		10.74 ^b^	12.35 ^b^	15.43 ^a^		11.00 ^b^	12.00 ^b^	17.06 ^a^	0.640	0.331	<0.01	0.380
IL-2 (pg/mL)	463.83		681.50 ^bc^	900.40 ^a^	984.27 ^a^		631.57 ^c^	844.24 ^ab^	1006.87 ^a^	48.55	0.485	<0.01	0.737
IL-6 (pg/mL)	71.66		106.43 ^c^	116.21 ^bc^	150.79 ^a^		110.53 ^c^	129.30 ^ab^	134.33 ^ab^	5.459	0.957	<0.01	0.057
IL-10 (pg/mL)	18.69		23.96 ^c^	32.35 ^ab^	38.69 ^a^		28.63 ^bc^	34.87 ^ab^	41.32 ^a^	2.090	0.059	<0.01	0.790
TNF-α (pg/mL)	125.42		128.60 ^b^	162.74 ^a^	176.43 ^a^		134.44 ^b^	160.49 ^a^	189.29 ^a^	6.860	0.331	<0.01	0.586

^1^ IgA = immunoglobulin A; IgG = immunoglobulin G; IgM = immunoglobulin M; C3 = complement component 3; C4 = complement component 4; IL-2 = interleukin 2; IL-6 = interleukin 6; IL-10 = interleukin 10; TNF-α = tumor necrosis factor-α. ^a–d^ Values with different superscript letters in the same row indicate significant differences (*p* < 0.05) and no shoulder notesindicated no significant differences (*p* > 0.05).

**Table 6:** Slaughter performance and meat quality of black goats at 30, 60, and 90 days of cadmium exposure.

Items	BAS		CON with Feeding Time, d				CAD with Feeding Time, d			SEM	*p*-Value		
	(0 d)		30	60	90		30	60	90		Diet	Time	Diet × Time
Dressing percentage (%)	44.51		36.96 ^b^	39.34 ^b^	46.17 ^a^		36.70 ^b^	38.67 ^b^	45.61 ^a^	1.022	0.555	<0.01	0.979
Loin muscle area (cm^2^)	5.76		3.08 ^b^	5.91 ^a^	6.23 ^a^		2.55 ^b^	6.01 ^a^	6.31 ^a^	0.488	0.775	<0.01	0.763
Color													
L*	42.34		43.36	38.34	35.45		42.07	38.04	36.23	2.432	0.893	0.026	0.914
a*	13.21		13.09	9.09	11.97		11.55	11.09	12.64	0.975	0.645	0.045	0.202
b*	6.95		7.11	5.04	5.43		6.41	5.09	6.18	0.574	0.941	0.021	0.460
Water loss rate (%)	8.64		10.13	7.59	10.09		9.76	5.3	8.18	1.435	0.205	0.052	0.782
Drip loss (%)	14.66		33.29 ^a^	31.12 ^a^	26.94 ^ab^		27.23 ^ab^	23.57 ^ab^	20.03 ^b^	2.310	<0.01	0.022	0.949
Cooking yield (%)	44.81		38.97 ^b^	48.01 ^a^	48.39 ^a^		38.99 ^b^	45.92 ^a^	43.58 ^ab^	1.352	0.047	<0.01	0.218
Shear force (kgf)	4.16		4.44 ^c^	12.04 ^a^	10.17 ^a^		4.94 ^c^	10.87 ^a^	7.96 ^b^	0.488	0.022	<0.01	0.031
pH_45min_	6.00		6.33 ^ab^	6.31 ^ab^	6.06 ^b^		6.52 ^a^	6.39 ^ab^	6.12 ^ab^	0.077	0.094	<0.01	0.659

^a–c^ Values with different superscript letters in the same row indicate significant differences (*p* < 0.05) and no shoulder notesindicated no significant differences (*p* > 0.05).

**Table 7:** Cadmium residues (μg/kg) in the organs and muscle of black goats at 30, 60, and 90 days of cadmium exposure.

Items	BAS		CON with Feeding Time, d				CAD with Feeding Time, d			SEM	*p*-Value		
	(0 d)		30	60	90		30	60	90		Diet	Time	Diet × Time
Heart	0.99		1.64 ^b^	1.24 ^b^	1.07 ^b^		7.89 ^a^	3.65 ^b^	3.78 ^b^	0.640	<0.01	<0.01	<0.01
Liver	10.68		12.46 ^b^	16.59 ^b^	31.18 ^b^		34.80 ^ab^	38.63 ^ab^	75.27 ^a^	9.484	<0.01	<0.01	0.422
Spleen	1.57		1.98 ^b^	4.33 ^b^	4.39 ^b^		15.72 ^b^	14.43 ^b^	38.48 ^a^	4.716	<0.01	0.017	0.034
Lung	10.17		9.28 ^bc^	7.68 ^c^	11.92 ^ab^		12.42 ^ab^	13.99 ^a^	12.63 ^ab^	0.854	<0.01	0.172	<0.01
Kidney	16.13		18.50 ^b^	40.64 ^b^	41.81 ^b^		31.21 ^b^	41.73 ^b^	78.67 ^a^	7.734	0.012	<0.01	0.078
Testis	1.54		1.39 ^c^	1.44 ^c^	1.57 ^c^		19.94 ^b^	26.53 ^b^	46.08 ^a^	4.040	<0.01	<0.01	<0.01
Lymph nodes	1.68		6.07 ^b^	7.49 ^b^	5.64 ^b^		32.30 ^b^	25.23 ^b^	102.78 ^a^	13.35	<0.01	0.013	0.010
Spine	2.11		21.25	20.67	21.23		25.95	22.75	24.00	1.397	<0.01	0.412	0.630
Skin	12.09		27.16	23.55	20.74		27.16	26.01	22.26	3.440	0.072	0.318	0.056
Picnic shoulder	1.94		8.58 ^b^	7.73 ^b^	23.25 ^b^		16.54 ^b^	18.07 ^b^	56.67 ^a^	4.401	<0.01	<0.01	0.012
Hindquarter	1.21		21.53 ^b^	18.45 ^b^	13.94 ^b^		33.88 ^b^	29.39 ^b^	46.70 ^a^	5.935	<0.01	0.565	0.147
Intercostal muscles	1.55		16.21 ^ab^	16.59 ^ab^	15.71 ^b^		22.05 ^a^	21.44 ^ab^	16.79 ^ab^	1.466	<0.01	0.102	0.246
Longissimus dorsi	1.10		11.31 ^b^	11.15 ^b^	12.02 ^ab^		17.49 ^ab^	24.71 ^a^	14.68 ^ab^	2.967	<0.01	0.284	0.190

^a–c^ Values with different superscript letters in the same row indicate significant differences (*p* < 0.05) and no shoulder notesindicated no significant differences (*p* > 0.05).

## Data Availability

The original contributions presented in the study are included in the article/Supplementary Materials; further inquiries can be directed to the corresponding authors.

## Supplementary Materials

The following supporting information can be downloaded at https://www.mdpi.com/article/10.3390/ani16193115/s1, Table S1: Normality test of Cadmium residues; Table S2: Normality test of serum biochemical indices; Table S3: Normality test of serum immune indices; Table S4: Normality test of growth performance, meat quality, and tissue accumulation; Table S5: Normality test of CdE.
