# Supplementary material for: Dietary Cadmium Exposure in Black Goats: Impacts on Growth Performance, Serum Biochemical and Immune Parameters, Meat Quality, and Tissue Accumulation
Source: Animals (Basel). 2026 Oct 4;16(19):3115. doi: 10.3390/ani16193115 (PMC13663565; doi:10.3390/ani16193115)
Supplement: Supplementary file 1 [file animals-16-03115-s001.zip › animals-4578342-supplementary.pdf]

Table S1. Normality test of Cadmium residues

Data category	Items	Sample range	Total number of records	SW statistic	p-value	SW Judgment
Cadmium residues	Heart	Including the Bas group	42	0.7506	4.570403E-07	Detect deviation from normality
Cadmium residues	Heart	Excluding the Bas group	36	0.7879	9.451837E-06	Detect deviation from normality
Cadmium residues	Liver	Including the Bas group	42	0.7212	1.343775E-07	Detect deviation from normality
Cadmium residues	Liver	Excluding the Bas group	36	0.7325	9.547823E-07	Detect deviation from normality
Cadmium residues	Spleen	Including the Bas group	42	0.6407	1.174011E-08	Detect deviation from normality
Cadmium residues	Spleen	Excluding the Bas group	36	0.6631	1.398237E-07	Detect deviation from normality
Cadmium residues	Lung	Including the Bas group	42	0.9447	4.190097E-02	Detect deviation from normality
Cadmium residues	Lung	Excluding the Bas group	36	0.9427	6.181978E-02	No deviation from normality was detected.
Cadmium residues	Kidney	Including the Bas group	42	0.8989	1.335616E-03	Detect deviation from normality
Cadmium residues	Kidney	Excluding the Bas group	36	0.9191	1.181219E-02	Detect deviation from normality
Cadmium residues	Testis	Including the Bas group	42	0.7326	2.139893E-07	Detect deviation from normality
Cadmium residues	Testis	Excluding the Bas group	36	0.7776	6.035619E-06	Detect deviation from normality
Cadmium residues	Lymph nodes	Including the Bas group	42	0.5696	6.766430E-10	Detect deviation from normality
Cadmium residues	Lymph nodes	Excluding the Bas group	36	0.5986	1.002560E-08	Detect deviation from normality
Cadmium residues	Spine	Including the Bas group	42	0.7929	3.129430E-06	Detect deviation from normality
Cadmium residues	Spine	Excluding the Bas group	36	0.9735	5.297936E-01	No deviation from normality was detected.
Cadmium residues	Skin	Including the Bas group	42	0.9614	1.659098E-01	No deviation from normality was detected.
Cadmium residues	Skin	Excluding the Bas group	36	0.9318	2.832991E-02	Detect deviation from normality
Cadmium residues	Picnic shoulder	Including the Bas group	42	0.7436	3.390374E-07	Detect deviation from normality
Cadmium residues	Picnic shoulder	Excluding the Bas group	36	0.7098	4.053486E-07	Detect deviation from normality
Cadmium residues	Hindquarter	Including the Bas group	42	0.8942	1.120779E-03	Detect deviation from normality
Cadmium residues	Hindquarter	Excluding the Bas group	36	0.8549	2.976739E-04	Detect deviation from normality
Cadmium residues	Intercostal muscles	Including the Bas group	42	0.9002	1.462628E-03	Detect deviation from normality
Cadmium residues	Intercostal muscles	Excluding the Bas group	36	0.9364	3.935155E-02	Detect deviation from normality
Cadmium residues	Longissimus dorsi	Including the Bas group	42	0.9000	1.441247E-03	Detect deviation from normality
Cadmium residues	Longissimus dorsi	Excluding the Bas group	36	0.8318	7.457041E-05	Detect deviation from normality

Table S2. Normality test of serum biochemical indices

Data category	Items	Sample range	Total number of records	SW statistic	p-value	SW Judgment
serum biochemical indices	ALB (g/L)	Including the Bas group	78	0.9562	9.075000E-03	Detect deviation from normality
serum biochemical indices	ALB (g/L)	Excluding the Bas group	72	0.9472	4.457043E-03	Detect deviation from normality
serum biochemical indices	A/G	Including the Bas group	78	0.9861	5.568050E-01	No deviation from normality was detected.
serum biochemical indices	A/G	Excluding the Bas group	72	0.9824	4.116085E-01	No deviation from normality was detected.
serum biochemical indices	LDL-C (mmol/L)	Including the Bas group	78	0.7469	2.806512E-10	Detect deviation from normality
serum biochemical indices	LDL-C (mmol/L)	Excluding the Bas group	72	0.9308	6.729226E-04	Detect deviation from normality
serum biochemical indices	TG (mmol/L)	Including the Bas group	78	0.8767	2.106346E-06	Detect deviation from normality
serum biochemical indices	TG (mmol/L)	Excluding the Bas group	72	0.8685	2.376198E-06	Detect deviation from normality
serum biochemical indices	HDL-C (mmol/L)	Including the Bas group	78	0.9291	3.150933E-04	Detect deviation from normality
serum biochemical indices	HDL-C (mmol/L)	Excluding the Bas group	72	0.9703	8.626653E-02	No deviation from normality was detected.
serum biochemical indices	GGT (U/L)	Including the Bas group	78	0.8546	3.491133E-07	Detect deviation from normality
serum biochemical indices	GGT (U/L)	Excluding the Bas group	72	0.8502	5.860260E-07	Detect deviation from normality
serum biochemical indices	ALT (U/L)	Including the Bas group	78	0.9655	3.880738E-02	Detect deviation from normality
serum biochemical indices	ALT (U/L)	Excluding the Bas group	72	0.9658	5.564316E-02	No deviation from normality was detected.
serum biochemical indices	AST/ALT	Including the Bas group	78	0.9169	1.030495E-04	Detect deviation from normality
serum biochemical indices	AST/ALT	Excluding the Bas group	72	0.9085	8.574264E-05	Detect deviation from normality
serum biochemical indices	AST (U/L)	Including the Bas group	78	0.8091	1.885279E-08	Detect deviation from normality
serum biochemical indices	AST (U/L)	Excluding the Bas group	72	0.7671	4.207139E-09	Detect deviation from normality
serum biochemical indices	CR (μmol/L)	Including the Bas group	78	0.9272	2.556866E-04	Detect deviation from normality
serum biochemical indices	CR (μmol/L)	Excluding the Bas group	72	0.9182	1.776197E-04	Detect deviation from normality
serum biochemical indices	ALP (U/L)	Including the Bas group	78	0.8992	3.192311E-05	Detect deviation from normality
serum biochemical indices	ALP (U/L)	Excluding the Bas group	72	0.8979	5.893644E-05	Detect deviation from normality
serum biochemical indices	GLU (mmol/L)	Including the Bas group	78	0.8801	2.472883E-06	Detect deviation from normality
serum biochemical indices	GLU (mmol/L)	Excluding the Bas group	72	0.9811	3.507282E-01	No deviation from normality was detected.
serum biochemical indices	BUN (mmol/L)	Including the Bas group	78	0.9807	2.847404E-01	No deviation from normality was detected.
serum biochemical indices	BUN (mmol/L)	Excluding the Bas group	72	0.9842	5.065470E-01	No deviation from normality was detected.
serum biochemical indices	UA (umol/L)	Including the Bas group	78	0.8671	8.260769E-07	Detect deviation from normality
serum biochemical indices	UA (umol/L)	Excluding the Bas group	72	0.8598	1.043744E-06	Detect deviation from normality
serum biochemical indices	GLB (g/L)	Including the Bas group	78	0.9495	3.742743E-03	Detect deviation from normality
serum biochemical indices	GLB (g/L)	Excluding the Bas group	72	0.9456	3.645169E-03	Detect deviation from normality
serum biochemical indices	TC (mmol/L)	Including the Bas group	78	0.8238	3.181943E-08	Detect deviation from normality
serum biochemical indices	TC (mmol/L)	Excluding the Bas group	72	0.9524	8.416022E-03	Detect deviation from normality
serum biochemical indices	TP (g/L)	Including the Bas group	78	0.9871	6.195332E-01	No deviation from normality was detected.
serum biochemical indices	TP (g/L)	Excluding the Bas group	72	0.9848	5.396808E-01	No deviation from normality was detected.

Table S3 Normality test of serum immune indices

Data category	Items	Sample range	Total number of records	SW statistic	p-value	SW Judgment
serum immune indices	IGA	Including the Bas group	78	0.9915	8.892184E-01	No deviation from normality was detected.
serum immune indices	IGA	Excluding the Bas group	72	0.9856	5.814014E-01	No deviation from normality was detected.
serum immune indices	IGG	Including the Bas group	78	0.9660	3.546047E-02	Detect deviation from normality
serum immune indices	IGG	Excluding the Bas group	72	0.9532	9.236456E-03	Detect deviation from normality
serum immune indices	C3	Including the Bas group	78	0.9839	4.312896E-01	No deviation from normality was detected.
serum immune indices	C3	Excluding the Bas group	72	0.9748	1.579148E-01	No deviation from normality was detected.
serum immune indices	C4	Including the Bas group	78	0.9806	2.794847E-01	No deviation from normality was detected.
serum immune indices	C4	Excluding the Bas group	72	0.9571	1.517386E-02	Detect deviation from normality
serum immune indices	IL-2	Including the Bas group	78	0.9777	1.866256E-01	No deviation from normality was detected.
serum immune indices	IL-2	Excluding the Bas group	72	0.9703	8.593138E-02	No deviation from normality was detected.
serum immune indices	IL-6	Including the Bas group	78	0.9855	5.225365E-01	No deviation from normality was detected.
serum immune indices	IL-6	Excluding the Bas group	72	0.9782	2.461595E-01	No deviation from normality was detected.
serum immune indices	IL-10	Including the Bas group	78	0.9782	2.022110E-01	No deviation from normality was detected.
serum immune indices	IL-10	Excluding the Bas group	72	0.9722	1.105549E-01	No deviation from normality was detected.
serum immune indices	TNF-α	Including the Bas group	78	0.9706	6.847009E-02	No deviation from normality was detected.
serum immune indices	TNF-α	Excluding the Bas group	72	0.9663	5.078966E-02	No deviation from normality was detected.
serum immune indices	IgM	Including the Bas group	78	0.9791	2.290798E-01	No deviation from normality was detected.
serum immune indices	IgM	Excluding the Bas group	72	0.9714	9.952834E-02	No deviation from normality was detected.

Table S4. Normality test of growth performance, meat quality, and tissue accumulation

Items	Sample range	Total number of records	SW statistic	p-value	SW Judgment
Dressing percentage (%)	Including the Bas group	42	0.9382	0.024650	Detect deviation from normality
Dressing percentage (%)	Excluding the Bas group	36	0.9353	0.036392	Detect deviation from normality
Loin muscle area (cm2)	Including the Bas group	42	0.9516	0.073977	No deviation from normality was detected
Loin muscle area (cm2)	Excluding the Bas group	36	0.9432	0.063818	No deviation from normality was detected
L*	Including the Bas group	42	0.9597	0.143796	No deviation from normality was detected
L*	Excluding the Bas group	36	0.9558	0.159516	No deviation from normality was detected
a*	Including the Bas group	42	0.9663	0.246445	No deviation from normality was detected
a*	Excluding the Bas group	36	0.9840	0.869350	No deviation from normality was detected
b*	Including the Bas group	42	0.9673	0.266235	No deviation from normality was detected
b*	Excluding the Bas group	36	0.9729	0.510244	No deviation from normality was detected
Water loss rate (%)	Including the Bas group	42	0.9211	0.006555	Detect deviation from normality
Water loss rate (%)	Excluding the Bas group	36	0.9167	0.010073	Detect deviation from normality
Drip loss (%)	Including the Bas group	42	0.9615	0.166715	No deviation from normality was detected
Drip loss (%)	Excluding the Bas group	36	0.9647	0.298249	No deviation from normality was detected
Cooking yield (%)	Including the Bas group	42	0.9474	0.052196	No deviation from normality was detected
Cooking yield (%)	Excluding the Bas group	36	0.9469	0.083525	No deviation from normality was detected
Shear force (kgf)	Including the Bas group	42	0.8869	5.991E-04	Detect deviation from normality
Shear force (kgf)	Excluding the Bas group	36	0.8995	0.003308	Detect deviation from normality
pH45min	Including the Bas group	42	0.9523	0.078217	No deviation from normality was detected
pH45min	Excluding the Bas group	36	0.9735	0.529291	No deviation from normality was detected
Body height (cm)	Including the Bas group	42	0.9697	0.323188	No deviation from normality was detected
Body height (cm)	Excluding the Bas group	36	0.9763	0.621304	No deviation from normality was detected
Body side length (cm)	Including the Bas group	42	0.9417	0.032706	Detect deviation from normality
Body side length (cm)	Excluding the Bas group	36	0.9614	0.237219	No deviation from normality was detected
Chest circumference (cm)	Including the Bas group	42	0.9365	0.021680	Detect deviation from normality
Chest circumference (cm)	Excluding the Bas group	36	0.9462	0.079124	No deviation from normality was detected
Abdominal circumference (cm)	Including the Bas group	42	0.9230	0.007597	Detect deviation from normality
Abdominal circumference (cm)	Excluding the Bas group	36	0.9432	0.063873	No deviation from normality was detected
Cannon circumference (cm)	Including the Bas group	42	0.9517	0.074017	No deviation from normality was detected
Cannon circumference (cm)	Excluding the Bas group	36	0.9295	0.024098	Detect deviation from normality
Testicular circumference (cm)	Including the Bas group	42	0.9785	0.602273	No deviation from normality was detected
Testicular circumference (cm)	Excluding the Bas group	36	0.9622	0.250824	No deviation from normality was detected
initial_BW	Including the Bas group	42	0.9475	0.052548	No deviation from normality was detected
initial_BW	Excluding the Bas group	36	0.9522	0.122271	No deviation from normality was detected
end_BW	Including the Bas group	42	0.9283	0.011349	Detect deviation from normality
end_BW	Excluding the Bas group	36	0.9535	0.135042	No deviation from normality was detected
daily gain	Including the Bas group	42	0.9373	0.023086	Detect deviation from normality
daily gain	Excluding the Bas group	36	0.9037	0.004316	Detect deviation from normality
Heart index	Including the Bas group	42	0.9141	0.003925	Detect deviation from normality
Heart index	Excluding the Bas group	36	0.8770	8.502E-04	Detect deviation from normality
Liver index	Including the Bas group	42	0.9793	0.635683	No deviation from normality was detected
Liver index	Excluding the Bas group	36	0.9662	0.331988	No deviation from normality was detected
Spleen index	Including the Bas group	42	0.9236	0.007945	Detect deviation from normality
Spleen index	Excluding the Bas group	36	0.8507	1.975E-04	Detect deviation from normality
Lung index	Including the Bas group	42	0.8684	1.852E-04	Detect deviation from normality
Lung index	Excluding the Bas group	36	0.8020	1.781E-05	Detect deviation from normality
Kidney index	Including the Bas group	42	0.9404	0.029497	Detect deviation from normality
Kidney index	Excluding the Bas group	36	0.9316	0.027954	Detect deviation from normality

Table S5. Normality test of CdE

Items	Sample range	Total number of records	SW statistic	p-value	SW Judgment
Heart	Excluding the Bas group	36	0.6776	1.283E-07	Detect deviation from normality
Liver	Excluding the Bas group	36	0.8921	0.002084	Detect deviation from normality
Spleen	Excluding the Bas group	36	0.7608	2.961E-06	Detect deviation from normality
Lung	Excluding the Bas group	36	0.7633	3.283E-06	Detect deviation from normality
Kidneys	Excluding the Bas group	36	0.8260	5.596E-05	Detect deviation from normality
Testis	Excluding the Bas group	36	0.8445	1.424E-04	Detect deviation from normality
Muscle	Excluding the Bas group	36	0.8514	2.046E-04	Detect deviation from normality
Feces	Excluding the Bas group	36	0.8051	2.062E-05	Detect deviation from normality
Other parts	Excluding the Bas group	36	0.7989	1.547E-05	Detect deviation from normality
